# Mechanical Forces, Nucleus, Chromosomes, and Chromatin

**DOI:** 10.3390/biom15030354

**Published:** 2025-03-01

**Authors:** Malgorzata Kloc, Jarek Wosik

**Affiliations:** 1 Transplant Immunology, The Houston Methodist Research Institute, Houston, TX 77030, USA; 2 Department of Surgery, The Houston Methodist Hospital, Houston, TX 77030, USA; 3 MD Anderson Cancer Center, Department of Genetics, The University of Texas, Houston, TX 77030, USA; 4 Electrical and Computer Engineering Department, University of Houston, Houston, TX 77204, USA; jarek@uh.edu; 5 Texas Center for Superconductivity, University of Houston, Houston, TX 77204, USA

**Keywords:** chromatin, chromosomes, nucleus, transcription, actin, microtubules, mechanical force, mechanotransduction, mechanosensing

## Abstract

Individual cells and cells within the tissues and organs constantly face mechanical challenges, such as tension, compression, strain, shear stress, and the rigidity of cellular and extracellular surroundings. Besides the external mechanical forces, cells and their components are also subjected to intracellular mechanical forces, such as pulling, pushing, and stretching, created by the sophisticated force-generation machinery of the cytoskeleton and molecular motors. All these mechanical stressors switch on the mechanotransduction pathways, allowing cells and their components to respond and adapt. Mechanical force-induced changes at the cell membrane and cytoskeleton are also transmitted to the nucleus and its nucleoskeleton, affecting nucleocytoplasmic transport, chromatin conformation, transcriptional activity, replication, and genome, which, in turn, orchestrate cellular mechanical behavior. The memory of mechanoresponses is stored as epigenetic and chromatin structure modifications. The mechanical state of the cell in response to the acellular and cellular environment also determines cell identity, fate, and immune response to invading pathogens. Here, we give a short overview of the latest developments in understanding these processes, emphasizing their effects on cell nuclei, chromosomes, and chromatin.

## 1. Introduction

Mechanically, a cell is a biological polymer gel whose state of gelling and mechanical properties depend on the dynamics of polymerization of G-actin (monomer) into F-actin (filament), microtubules, intermediate filaments, and their crosslinking and connection to the molecular motors, which makes them contractile [1]. Cell cytoskeleton not only fulfills the mechanical needs of cells by supporting cell morphology, movement, division, and intracellular transport vesicles and organelles but also participates in the immune response to invading pathogens. Bacteria and viruses can manipulate the host cell cytoskeleton to ensure their intracellular entry, survival, and replication [2,3,4,5,6,7,8]. For example, viruses reorganize the actin cytoskeleton underlying the cell membrane aiding endocytosis-based entry into the cell. They can also hijack microtubular motor proteins for movement to replication sites or to the cell periphery for exit. They can change the organization and expression of cytoskeletal components to create an environment favorable for replication. They promote disaggregation of nuclear lamina, therefore allowing virus entry into the nucleus for replication and subsequent egress of the progeny [9,10]. Some baculoviruses use the mechanical force created by the intranuclear actin filament-based protrusions for the nuclear envelope disruption and egressing from the nucleus [11,12]. Many species of bacteria (e.g., *Listeria monocytogenes*, *Shigella flexneri*, *Burkholderia* spp. and *Rickettsia* spp.) polymerize actin filaments of the host cells to produce actin “comets” on their surface, which propel bacteria through the cytoplasm and facilitate intercellular spreading [13].

The mechanical state of cell cytoplasm transmits to the nucleus, nucleoskeleton, and intranuclear components through the specialized structures and molecules of the nuclear membrane and perinuclear cytoskeleton [14,15,16,17,18]. Although the cell nucleus is the most rigid among all organelles, it can also reversibly deform and reposition in response to mechanical stress and substrate stiffness. Recent studies showed that nuclear remodeling and change in its stiffness depends on the intranuclear motor protein, the Brahma-related gene 1 (BRG1) ATPase, which belongs to the family of ATP-dependent chromatin remodeler SWI/SNF (switch/sucrose nonfermenting) enzymes [19,20], and a dynamic pool of heterochromatin Protein1/chromatin-associated protein swi6 (HP1a/Swi6) [21,22]. One of the novel methods instrumental in assessing tension dynamics is the tension gauge tethers (TGTs), short segments of double-stranded DNA (dsDNA) that irreversibly break in response to sheer-stretch tension. TGTs are attached to the tension receptors, such as T-cell receptors, cadherins, or integrins and assess if the duration of applied tension is sufficient to activate mechanotransduction responses and if the tension level surpasses the activation threshold [23,24,25,26]. Another novel method is the electrochemical DNA-based force sensors (a tension gauge tether and DNA hairpin) for measuring piconewton-scale forces generated during cell adhesion [27]. Unfortunately, the DNA sensors are vulnerable to deoxyribonucleases (DNases). To overcome this, a new class of tension sensors was constructed from peptide nucleic acid (PNA) and DNA. These hybrid sensors are not only resistant to DNases but also have the highest signal-to-noise ratio and specificity. Thus, they can be used to study mechanobiology in cells expressing DNases [28]. There are also efforts to recreate in vitro mechanical properties of adherent cells and analyze how the integrin-based focal contacts interact with extracellular matrix (ECM) and transmit stresses generated by actomyosin contraction to ECM by encapsulating cortical actomyosin networks in lipid membrane vesicles adhered to various substrates [29]. Recent studies on the effect of magnetically driven hydrogel substrate on the macrophages showed that the dynamic stiffness combined with the pulsed magnetic field pushes macrophages toward the anti-inflammatory (M2) phenotype [30]. Additionally, a recently created computational model of the effects and forces involved in mechanical cyclic stretching of cells on a flexible membrane [31] may be a new tool for interpreting the results of many mechanotransduction experimental models [32,33], while recently developed single-molecule force spectroscopy and molecular tweezer techniques measuring the compaction of DNA and manipulation with piconewton-scale forces may help in understanding the dynamics of DNA organization and mechanical parameters of nucleoprotein filaments [34].

## 2. Mechanical Forces and Cell Nucleus

The cell nucleus responds not only to external cellular and acellular mechanical stimuli but also to mechanical signals generated intracellularly during cell migration, differentiation, and cell division. The nucleus contains the nucleoskeleton with a pool of nuclear G- and F-actin that responds to mechanical stimuli and regulates the intranuclear movement, transcription, chromatin remodeling, and DNA repair [12,16,35,36] and tethers chromatin to the nuclear membrane [37]. Nuclear G-actin polymerizes in response to DNA damage, and resulting actin filaments relocate chromatin regions containing double-strand breaks to the repair sites at the nuclear periphery [38]. The nucleus also acts as a mechanosensor, and nuclear deformations perceived by the nucleoskeleton cause conformational changes in chromatin, rearrangement of genes, and, thus, modulate transcription [39]. The following paragraphs describe how the nucleus responds to various extra- and intracellular mechanical cues and the reciprocal effects of nuclear mechanoresponses on cell functions and regulation of gene activity.

### 2.1. Nuclear Positioning

The position of the nucleus within the cell depends on the cell type, differentiation, migratory behavior, phase of the cell cycle, and type (symmetric versus asymmetric) of cell division [40]. For example, in mammalian columnar and secretory epithelia and skeletal muscle, the nuclei are located asymmetrically (either apically or basally in epithelia or peripherally or centrally in muscle). In contrast, squamous and cuboidal epithelia, chondroblasts, osteoblasts, and cardiac muscle cells have centrally located nuclei. The central position of the nucleus may change when the cell starts to move or secrete. In developing vertebrate retina, the neuroepithelial nuclei migrate along the apical–basal axis, depending on the cell cycle phase. In the M-phase, nuclei are at the apical surface, while during the G1-, S-, and G2-phases, they move basally [41,42]. In early mouse embryos, the position of the nucleus within the blastomere determines the plane of cell division. Blastomeres with basally located nuclei divide asymmetrically, while those with apically located nuclei divide symmetrically [43]. Studies from our laboratory showed that the force created by magnetic field gradient, or genetic or pharmacologic inhibition of the actin cytoskeleton regulator, the RhoA/ROCK pathway, affects actin cytoskeleton distribution and polymerization in macrophages, resulting in macrophage elongation and repositioning of the nucleus from the front to the tail [44,45,46,47]. Because the position of the nucleus is crucial for cell function and fate, nuclear mispositioning may lead to changes in cell fate and various pathologies, including cancer and skeletal muscle diseases, such as centronuclear and dilated myopathy and Emery–Dreifuss muscular dystrophy [48,49,50,51,52].

### 2.2. Nuclear Drilling as a Driving Force of Transmigration Through the Endothelial Barrier

Usually, in migrating cells such as fibroblasts, mesenchymal cells, neurons, and cancer cells, the nucleus is positioned away from the cell leading edge by a retrograde flow of actin mediated by myosin and Cdc42. In some cells, such as leukocytes transmigrating through the endothelial barrier, the nucleus becomes positioned in the cell front. Once there, it forms the lobes, which are incorporated, using myosin II, into the frontal lamellipodia. These lobes reversibly disassemble actin filaments between stress fibers in the endothelial barrier, bend stress fibers, and generate micron-size intercellular gaps, allowing leukocytes to drill and squeeze through the endothelial barrier [53]. Squeezing through the narrow spaces and cell spreading not only deforms the cell but also its nucleus. Mechanical deformation of the nucleus during the squeezing through the narrow spaces or mechanical compression during development increases DNA replication stress (probably through replication fork stalling) resulting in deformation-induced DNA damage and activation of the DNA damage response [54].

### 2.3. Nuclear Shape Changes During Cell Spreading

Studies of the mechanical forces behind the changes in nucleus shape during cell spreading showed that in spreading fibroblasts, the nuclei flatten during an early stage of spreading but with time, the nuclei round up and their dimensions stabilize. Inhibition of actin polymerization and myosin light chain kinase limits flattening of nuclei and inhibition of myosin-II ATPase eliminates nuclear rounding. The authors proposed a computational model of how frictional transmission of stress from the moving cell boundaries to the surface of the nucleus drives its shape changes during spreading [55]. Another study showed that cell and nuclear compression causes reinforcement of microtubules at the rear of the nucleus, creating the mechanostat structure that facilitates positioning of the nucleus and organizes the contractility events in space and time [56].

### 2.4. Nuclear Mechanosensing and Mechanotransduction

**Figure 1:**
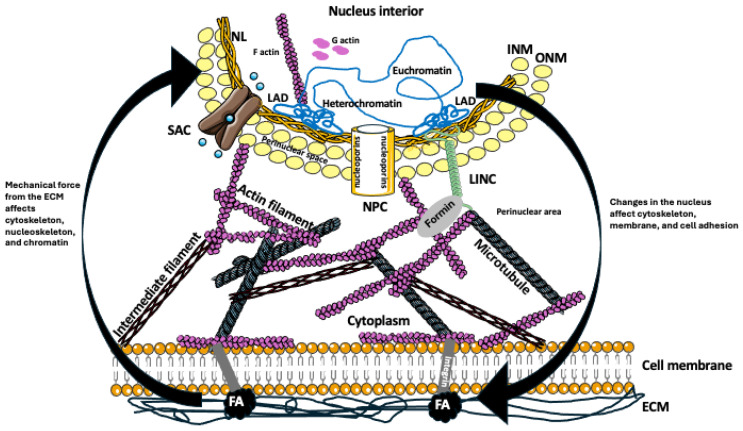
Components of the mechanotransduction pathways. Highly simplified illustration of mechanotransduction pathway between the extracellular substrate, cellular membrane, and the nucleus. Focal adhesions (FAs) dictate the strength of cell attachment to the extracellular matrix (ECM). One of the components of FA, the transmembrane protein integrin, physically connects ECM to the cell cytoskeleton composed of intermediate filaments, actin filaments, and microtubules. Any mechanical-force-induced change at the cell membrane (e.g., stretching, pulling, tension, compression, etc.) is transmitted (indicated by the arrow) to the underlying cytoskeleton. The cytoplasm near the nucleus (the perinuclear area) contains the perinuclear cytoskeleton that, via the actin cytoskeleton remodelers, the formin proteins, is connected to the LINC complexes. LINCs are the assemblies of several proteins spanning the nuclear envelope and physically connecting the perinuclear cytoskeleton to the nuclear lamina (NL) underlying the nuclear membrane. The NL associates with heterochromatin (condensed chromatin) through the lamina-associated domains (LADs), aiding in the functional organization of the genome and separating heterochromatin from the transcriptionally active (decondensed) euchromatin. The nuclear envelope contains an outside (ONM) and internal (INM) membrane separated by the perinuclear space which facilitates selective transport of RNA and proteins across the nuclear membrane. The ONM is connected to the perinuclear endoplasmic reticulum, whose lumen is contiguous with the perinuclear space. The nuclear envelope is penetrated by the mechanosensitive channels and pores. Stretch-activated ion channels (SACs) open in response to the nuclear membrane stretching and regulate the flow of ions across the membrane. Nuclear pore complexes (NPCs), lined with nucleoporins, shrink, or dilate in response to mechanical force and regulate the transport of RNA and proteins to and from the nucleus. Additionally, the cell nucleus contains a pool of intranuclear G-actin and F-actin (filaments), which participate in mechanosensing, mechanoresponse, chromatin remodeling, DNA repair, and transcription. Arrows indicate the transmission of mechanical signals from the cell surface to the nucleus and signals from the nucleus to the cell surface.

Like a whole cell, the nucleus and interconnected endoplasmic reticulum can sense a deformation or strain originating from the expansion or shrinking of the cell, changes in nuclear positioning, cell squeezing through the narrow spaces, cell migration, sheer flow of body fluids, and deformation of extracellular matrix. These mechanical forces may open stretch-activated ion channels (SACs) in the nuclear membrane or change the conformation of mechanosensitive proteins that, in turn, will affect nuclear transport and chromatin structure, its distribution, and transcriptional activity (Figure 1) [14,57,58,59,60,61]. Computational and experimental modeling of the nuclear strain distribution showed that different regions of the nucleus have different mechanical properties and responses to mechanical stretching [62]. Additionally, the mechanical force can shrink or dilate the diameter of nuclear pore complexes (NPCs). NPCs are mechanosensitive cylinders embedded in the nuclear membrane, lined with 30 different nucleoporins (which can bind to transport factors), and regulating the transport of RNA and proteins to and from the nucleus (Figure 1) [63,64,65]. The outer and inner nuclear membrane are spaced by the perinuclear space, which should not be confused with a perinuclear area in the vicinity of the nucleus (Figure 1). The outer nuclear membrane (ONM) is surrounded by perinuclear actin filaments, actomyosin, and microtubules, which regulate nuclear shape and counteract deformation [58]. Actin filaments are anchored at the nuclear membrane by TAN (Transmembrane Actin-associated Nuclear) lines composed of the linker of nucleoskeleton and cytoskeleton (LINC) complex containing Nesprin-1/2, Sad1/UNC-84 (SUN) domain-containing SUN protein, and Klarsicht/ANC-1/Syne-1 homology (KASH) domain-containing KASH protein, which are anchored at nuclear lamina, allowing nuclear movement by actin retrograde flow [49,50,66,67,68]; (Figure 1). The SUN traverses the inner and KASH the outer nuclear membranes. SUN and KASH interact with the nuclear lamina and actin/microtubule cytoskeleton and physically connect the nucleus to the other parts of the cell [69,70]. Interestingly, recent studies showed that this three-dimensional perinuclear network of the cytoskeleton is also involved in the assembly of biomolecular condensates and orchestrates the movement of molecules within the perinuclear area [71] (Figure 1). The nuclear condensates compartmentalize nuclear functions, concentrate macromolecules relevant to gene expression, and together with the nuclear lamina regulate the mechanical properties of the nucleus (Negri et al., 2023) [72].

The inner nuclear membrane (INM) is covered by nuclear lamina (NL), a network of mainly type V intermediate filament proteins (lamins) [73,74]; (Figure 1). NL mechanically supports nuclear architecture and contacts hundreds of chromatin regions containing several thousand genes. The mutations in nuclear lamins (so-called laminopathies) alter nuclear shape and cause many diseases such as muscular dystrophies, familial partial lipodystrophy, dilated cardiomyopathy, and Hutchinson–Gilford progeria syndrome (HGPS) [58,74,75,76,77]. In progeria, the aberrantly spliced and improperly farnesylated nuclear lamin A (progerin) cannot be properly incorporated into nuclear lamina, causing changes in the distribution and level of heterochromatin, defects in NPCs and mechanotransmission, and shortening telomeres [75,76,77]. Interestingly, the mechanical stability of telomeres, which are different from the rest of chromatin due to their repetitive sequences, depends on the telomere repeat binding factor 2 (TRF2), a component of shelterin (telosome) protein complex that protects telomeres from DNA repair and regulates telomerase activity [78,79].

The lamina-associated domains (LADs) that cover approximately one-third of the total genome participate in establishing interphase chromosome architecture and spatial organization of the genome and repress gene transcription. Since LADs contain repressed chromatin, they are enriched in heterochromatin-associated histone modifications such as H3K9me2,3 which is recognized by the chromodomain of heterochromatin protein 1 (HP1) that regulates heterochromatin formation and gene repression [21,61,80,81]. Because the nuclear membrane and NL are physically connected to the perinuclear and cytoplasmic cytoskeleton (actin filaments and microtubules) by the LINC complex, any mechanical deformation of the cell membrane and cytoplasm, as well as substrate stiffness and tension derived from the focal adhesions, is transmitted to the nucleus. Focal adhesions contain transmembrane integrins and intracellularly located Paxillin, Talin, Vinculin, Src, FAK, and CAS, which physically connect the extracellular matrix to the network of intracellular actin filaments and nucleus (Figure 1) [1,82]. Studies in the fibrosis model showed that mechanical stress of progressive scarring causes nuclear softening and increases chromatin accessibility through the loss of H3K9Me3 and the serine/threonine-protein kinase P21-activated kinase PAK1 mechanosensing, β1 integrin-dependent, pathway [83].

Other proteins involved in the mechanoresponses of the nucleus, through the coupling of perinuclear actin to the nucleus, are the formin homology domain-containing (FHOD) formins. They have F-actin bundling activity regulated by ROCK, Src, and ERK1/2 kinases. FHODs associate with the outer nuclear membrane by binding their amino termini to the spectrin repeats (SRs) in Nesprin-1G/2G, a component of LINC, transmitting mechanical information to the nucleus [84]. Studies of nuclear deformation during directional cell stretching on the substrate showed that the lateral compressive forces exerted by the lateral actin cytoskeleton are crucial for the orientation-dependent deformation of the nucleus and mechanosensing responses to the direction of stretching [85]. Additionally, super-resolution and live imaging showed that the replication stress induces nuclear F-actin polymerization that, in turn, increases nuclear volume and sphericity, counteracting the deformation of the nucleus. Filamentous actin, together with myosin II, moves the stressed-replication foci to the nuclear periphery, promoting replication stress repair and suppressing chromosome and mitotic aberrations [86,87,88]. Thus, the mechanical properties of the perinuclear and nuclear cytoskeleton, nuclear lamina and chromatin are critical for response and adaptation to internal and external mechanical forces [89,90,91].

## 3. Mechanical Forces and Chromosomes

During cell division, chromosomes are constantly exposed to mechanical forces, such as pulling by the microtubules of the mitotic spindle and changes in the shape of the nucleus [92]. Resistance of chromosomes to mechanical stretching is indispensable for preserving their integrity. Recent studies using micromanipulation, stretching response, and molecular dynamic simulations to quantitatively describe the physical state of chromosomes during different phases of the cell cycle indicate that chromosomes are viscoelastic solids that behave like liquid-like (viscous) objects during interphase and like solid-like objects during cell division. Such changes in chromosome stiffness occur through the lengthwise compaction of the initially open structure. Additionally, chromosomes have linear elasticity, returning to the original shape even after being lengthwise stretched up to five times [93,94]. In preparation for cell division, the chromatin of the interphase nucleus is condensed approximately a thousandfold into rod-shaped individual chromosomes [95,96]. One of the existing models of mitotic chromosome postulates that a condensed chromosome consists of chromatin loops arranged into a spiral staircase attached to the central proteinaceous scaffolding, forming a “bottlebrush” structure [96]. Another model describes a chromosome as a heterogeneous aggregate of worm-like chains with nonlinear mechanical stiffening properties. Such nonlinear mechanics, by limiting deformation to the individual elements, is probably beneficial for maintaining the integrity of the whole chromosome. In both models, condensin I and II and topoisomerase IIα (TOP2A) participate in chromosome condensation [97,98]. Recent mapping of molecular densities around condensing and decondensing chromosomes showed that the density of various molecules, including RNAs and proteins in chromosome surroundings, increases from prophase to anaphase and decreases in telophase. These findings indicate that the transient changes in molecular density and the attractive forces between large structures in a crowded chromosomal milieu are also important for chromosome condensation [99]. On a similar theme, Hernandez-Armendariz et al. [100] showed that chromosome clustering during cell division exit is facilitated by the formation of a liquid-like layer on the chromosome surface. The main player in this process is the proliferation marker Ki-67 protein. During interphase, Ki-67 is in the nucleolus. At the mitotic entry, Ki-67 undergoes phosphorylation and forms a surfactant layer of brush-like structures on the surface of chromosomes, facilitating chromosome repulsion, dispersion, and connection to the mitotic spindle [101]. During mitotic exit in anaphase, Ki-67 dephosphorylates, and the brushes dissipate, allowing chromosome clustering within the future telophase nuclei. Thus, the phospho-switch transforms Ki-67 from a chromosome repellent to a chromosome attractant [100].

The separation of chromosomes during cell division is driven by the microtubules of the spindle. Chromosomes are connected to the microtubules by kinetochores, composed of about 100 different proteins, which transmit mechanical forces generated by molecular motors and microtubules to the chromosomes. The kinetochore-microtubule attachment, facilitated by the Astrin-SKAP kinetochore complex [102], faces two main mechanical challenges: it must resist the pulling force, making the attachment stable and be flexible enough to move chromosomes toward the microtubule plus ends. Recent mutational, laser ablation, and micromanipulation studies showed that kinetochore SKAP binds to microtubules with a lower resistance to sliding than other microtubule-binding molecules of the kinetochore, reducing kinetochore friction, making the attachment stronger and more stable under force. The authors propose that the key to the strong attachment under force is the mechanically heterogeneous interface, made of kinetochore proteins with both high and low sliding friction on microtubules [103].

### Mechanoregulation of Chromatin and Epigenetic Landscape

Any mechanical force effects on the cellular membrane and underlying cytoskeleton can be transmitted to chromatin, changing its architecture, structure, epigenetic features, and gene transcription [104,105,106,107,108,109]. In the 1940s, British embryologist and geneticist Conrad Waddington coined the concept of “epigenetic landscape” to describe how the plasticity of cellular phenotype affects epigenetic regulation and changes [110]. Epigenetic regulation involving chemical modifications of chromatin, such as histone modifications and DNA methylation status, alter gene transcription and cell plasticity. Epigenetic modifications together with a spatial organization of chromatin create a three-dimensional landscape within the nucleus. The epigenetic landscape is cell-type specific and can change in the disease and in response to mechanical cues [110,111].

**Figure 2:**
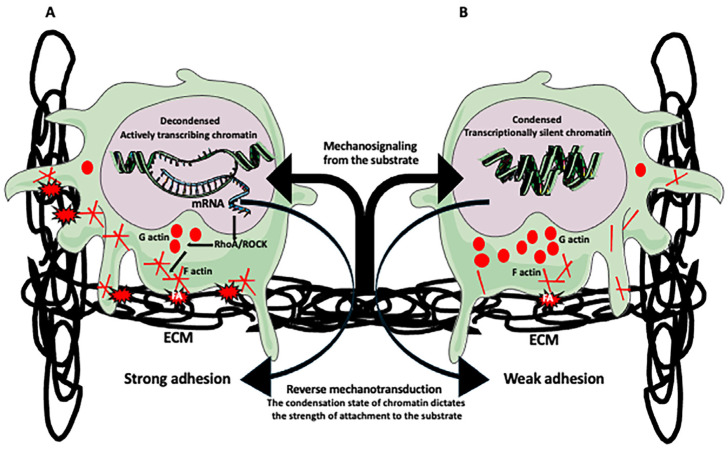
Reverse mechanotransduction. Mechanical forces created by an acellular (extracellular matrix, ECM) or cellular environment affect the cell cytoskeleton, which transduces signals to the nucleus, altering chromatin condensation and, thus, its transcriptional activity. Decondensed chromatin (euchromatin) is transcriptionally active, and condensed chromatin (heterochromatin) is either silent or has a low transcription activity. In the so-called reverse mechanotransduction, the state of chromatin condensation dictates the strength of cell adhesion to the extracellular substrate. (**A**) Decondensed chromatin transcribes mRNAs from various mechanically relevant signaling genes. These mRNAs are translated into signaling proteins, such as GTPase RhoA and its effector kinase Rock, or co-factors regulating their activity. Increased activity of the Rho/Rock pathway polymerizes globular (G) actin into actin filaments (F-actin), stimulating the formation of focal adhesions (FAs), which creates a strong attachment of the cell to the substrate. (**B**) Transcriptionally silent (highly condensed) chromatin results in the low level and low activity of the mechanosignaling pathway components, in consequence, decreasing actin polymerization and production of FAs. A low abundance of FAs causes weak attachment to the substrate.

Chromatin has two main states: transcriptionally active and less compacted euchromatin and highly compacted and transcriptionally repressed heterochromatin. Recently developed methods, such as interferometric scattering correlation spectroscopy, will help in understanding the dynamics of chromatin condensation [112]. Simulations based on confocal microscopy data identified three distinct intranuclear mechanical phases: mRNA-rich interchromatin region, euchromatin, and heterochromatin, which respond differently to the externally applied extracellular matrix shear deformation [113]. While euchromatin is usually located near the center of the nucleus, heterochromatin contacts in the nuclear lamina layer underlying the inner nuclear envelope and through the LINC complexes (see Section 2.3) communicates with perinuclear and cellular cytoskeleton (Figure 1) [69]. One of the LINC components, the SUN2 protein, responds to the contractility of actomyosin, and disrupting its binding activity to NL and actin cytoskeleton causes uniform, instead of perinuclear, distribution of heterochromatin marker H3K9me3 that represses repetitive elements and non-coding parts of genes and silences lineage-inappropriate genes [114,115]. Besides affecting chromatin compaction and distribution, mechanical stress can cause changes in DNA, affecting the accessibility of different chromatin domains to transcription and other regulatory factors [69,111,116,117]. Studies showed that ataxia telangiectasia mutated (ATM) and ataxia telangiectasia and Rad3-related (ATR) DNA damage response (DDR) kinases that contain HEAT (Hungtinton, Elongation Factor 3) repeats with elastic properties, respond to mechanical stress. Following cytoskeleton stress, the ATM located on the actin cytoskeleton, promotes, via reactive oxygen species (ROS)-dependent pathway, phosphorylation of cytoskeleton and chromatin regulators, and remodeling of cytoskeleton and chromatin [33,118,119]. In reverse, DNA damage causing ATM-dependent changes in heterochromatin affects nuclear stiffness and shape and may lead to its rupture [120]. The mechanical functions of ATR at the nuclear envelope are to conduct mechanical signals from cytoskeleton to the nuclear envelope and regulate envelope–chromatin association and chromatin activity [121,122]. Interestingly, in a reverse mechanotransduction pathway, the chromatin condensation status and remodeling are transmitted from the nucleus to the cell surface, affecting, through the RhoA/ROCK pathway, the cell shape and strength of adhesion to the substrate (Figure 2); [123]. Recent studies showed that chromatin can change its mechanical state to protect genome integrity during cell and nucleus deformation. Calcium-dependent loss of H3K9me3-marked heterochromatin and nuclear softening counteract nuclear deformation caused by mechanical stretching and insulate the genome from mechanical force [124].

## 4. Mechanomemory

Mechanomemory (mechanical memory) is the ability of a cell to remember the effects of a mechanical stimulus long after its cessation [15,125]. One of the molecules involved in storing time- and force-dependent mechanical information are cytoskeletal linker proteins talins whose 13 α-helical bundles contained within the C-terminal rod domain function as force-dependent switches [126]. Also, the nucleus and chromatin can memorize mechanical responses even to short-lasting mechanical stress, for minutes/hours, or even many cell generations after stress cessation [111,127,128]. Experiments with direct chromatin stretching by ferromagnetic nanoparticles inside the nucleus or the magnetic beads at the cell surface showed that the increase in diffusivity of chromatin, nucleoplasm, and RNA polymerase II (RNA Pol II), RNA Pol II activity, and its co-dependent gene expression, lasted a long time after stress discontinuance. These results may explain a sustained upregulation of transcription observed in cells tens of minutes after force cessation [129]. Interestingly, RNA Pol II transcriptional activity generates a mechanical force of ten piconewtons, which affects loci dynamics and shapes the coexistence of fluid- and solid-like properties within chromatin [130]. Studies of stem cells grown on different stiffness substrates showed that mechanical memory of substrate properties is stored in the intracellular rheostat YAP/TAZ containing Yes-associated protein (YAP) and transcriptional coactivator with PDZ-binding domain (TAZ), and the pre-osteogenic transcription factor RUNX2 [131]. Recent studies using a fluorescent sensor of nucleocytoplasmic transport (Sencyt) in monolayers of epithelial and mesenchymal cells showed that YAP senses cellular density but is not affected by nuclear tension, deformation, and solidity that affect the nucleocytoplasmic transport of transcriptional regulators [132]. At the nuclear/chromatin level, the mechanical memory becomes stored through structural remodeling of chromatin architecture and trimethylation status of Histone H3 lysine 9 (H3K9). For example, the response of cells to the stiffness of extracellular matrix, compression, and hydrostatic pressure, down- or upregulates chromatin modifiers such as histone acetyltransferase (HATs), histone deacetylases (HDACs), and acetyl-CoA, changing spatial organization of genes and the transcription [125,133,134]. A histone that couples cell stiffness to chromatin compaction is the linker histone H1.0, which not merely turns genes on or off but also influences a gene-specific deposition of histone H3K27Ac, a mark of gene and enhancer activation and is involved in the formation of supra-nucleosomal chromatin higher-order structures [135,136].

Because the first target of mechanical forces created by the cellular and acellular environment is the cell membrane and underlying cortical actin cytoskeleton, it is not surprising that mechanical memory is stored in the actin cortex first. Cells migrating through the constricted spaces retain a long-term memory of shape, imposed by past confinement, through actin cortex remodeling and RhoA/ROCK pathway activity. The thickness of the cortex in compacted cells (in confined spaces) is double that in elongated cells (in open spaces), which suggests that the thickening of the cortex may serve as a mechanical memory of past confinement, allowing the cell to maintain compacted shape after moving into an open space. The mechanical memory of the previous morphological state is crucial for cells traversing a heterogenous space with successive areas of confinements and openings, allowing cells to retain a compacted shape during temporary unconfinement without pausing to reorganize their shape each time they encounter an opening [137]. Studies of astrocytes exposed to short pulses of fluid sheer stress showed that the development of prolonged mechanomemory requires an intact F-actin cytoskeleton [138]. Recent studies on cardiac fibroblasts (CFs) and cardiomyocytes (CMs) showed that cytoskeletal regulators of mechanical memory in response to the extracellular matrix stiffening are cell-type-specific and depend on actin filaments (in CFs) or microtubules (in CMs) [139]. Although outside the scope of this review, it should be mentioned that the actin and microtubule cytoskeleton and their regulatory proteins are also involved, via regulation of synaptic transmission and neuronal morphology, in forming and sustaining neuronal memory in the brain [140,141]. Although so far there is no direct proof, the mechanomemory may also depend on the viscoelastic rheological properties of the cytosol, i.e., its ability to behave like a viscous fluid and an elastic solid, the property that influences cell adhesion, migration, and differentiation [142,143].

Mechanical memory can create a potential problem for the therapeutic use of cells propagated in vitro for tissue regeneration procedures. For example, chondrocytes expanded for several generations on the 2D stiff substrates retain epigenetic memory of stiffness after transfer to a 3D in vivo environment [134], which may decrease their therapeutic effectiveness. Recent studies showing that chromatin and nucleoplasm retain the mechanomemory in protein diffusivity tens of minutes after the external force cessation [128] support the notion that mechanomemory may be extremely important for the development of various diseases and/or change the therapeutic outcome.

## 5. Concluding Remarks

Although every day brings discoveries in mechanosensing, mechanotransduction, and mechanomemory areas, we are still very far from understanding the details of these processes at cellular and molecular levels. The findings that a cell can remember previously encountered stresses and mechanical cues of cellular and acellular environment and, in response, can alter morphology, behavior, gene expression, and cell fate may change our understanding of many diseases, modify current therapeutic approaches, and help design novel therapies. Focused mainly on mammalian and human cells, our review omits many fascinating examples of mechanotransduction in various invertebrate and vertebrate species, including those in aquatic and/or colonial organisms. A comprehensive description of mechanisms of sensing and responding to the mechanical forces created by water current, movement, and pressure in aquatic animals and the transmission of mechanical information between the individuals within a colony would require a separate review.

## Data Availability

Not applicable.
